# Neonatal Cardiac and Respiratory Arrest During Linezolid Therapy: A Case Report

**DOI:** 10.7759/cureus.69480

**Published:** 2024-09-15

**Authors:** Weijuan Song, Jingjing Qi, Shuqi Fan, Junjun Xiao, Ming Li

**Affiliations:** 1 Pharmacy, The Fifth Affiliated Hospital of Zhengzhou University, Zhengzhou, CHN; 2 Pharmacy, Xiayi County Traditional Chinese Medicine Hospital, Shangqiu, CHN; 3 Pharmacy, Tangyin County People's Hospital, Anyang, CHN; 4 Pharmacy, Luohe Central Hospital, Luohe, CHN; 5 Pharmacy, The First People's Hospital of Shangqiu City, Shangqiu, CHN

**Keywords:** acidosis, cardiac respiratory arrest, case report, linezolid, neonate, sudden infant death syndrome

## Abstract

Lactic acidosis is one of the severe adverse reactions of linezolid. Its clinical manifestations are non-specific, primarily including abdominal discomfort, nausea, vomiting, diarrhea, weakness, lethargy, rapid breathing, and tachycardia, with no reports of cardiac and respiratory arrest. In this case report, we present a 13-day-old male infant with omphalitis caused by methicillin-resistant *Staphylococcus aureus* (MRSA) infection, who was treated with linezolid. He had lactic acidosis before treatment, which was not severe and was likely related to the infection. After linezolid therapy, he experienced cardiac and respiratory arrest, and re-measurement showed an increase in lactate levels. After resuscitation, linezolid withdrawal, and symptomatic treatment, lactate levels decreased. However, due to hypoxic-ischemic encephalopathy and uncorrectable ventricular arrhythmia caused by post-cardiopulmonary resuscitation myocardial damage, the infant died. A comprehensive autopsy and genetic testing were performed after death, and no congenital diseases or inherited metabolic diseases were found. Given that this case was a sudden infant death without typical symptoms of lactic acidosis and linezolid is often mistakenly considered safer than vancomycin in the treatment of special populations, this paper analyzes and discusses this to draw attention to clinical treatment. More research is needed in the future to fully demonstrate its causal relationship and mechanism of action.

## Introduction

Linezolid is an oxazolidinone class of antimicrobial agents, and lactic acidosis is one of its serious adverse reactions, which has been reported on the drug's label and in both marketing experiences [1,2], with an incidence rate of 4.4%-6.8% [3,4]. The clinical manifestations are non-specific, primarily including abdominal discomfort, nausea, vomiting, diarrhea, weakness, lethargy, rapid breathing, and tachycardia. Lactic acidosis can occur 1-16 weeks after the use of oxazolidinone drugs [2,5] and is commonly seen in long-term treatments (40-50 days) [6]. The mortality rate associated with oxazolidinone-induced lactic acidosis is 25%-50% [2,7]. Once detected, oxazolidinone drugs should be immediately discontinued, and lactate levels may return to normal within 2-14 days [6]. Liver dysfunction, kidney dysfunction, mitochondrial DNA A2706G polymorphism, and the concurrent use of drugs affecting mitochondrial function all increase the risk of lactic acidosis. Persistent hyperlactatemia is associated with a poorer prognosis [3]. There is a dose-response relationship between lactate levels and mortality. An initial blood lactate level exceeding 2.5 mmol/L is significantly correlated with increased 28-day mortality [8]. Lactic acidosis is generally treated symptomatically with supportive measures such as oxygen therapy, fluid resuscitation, vasopressors, and blood purification. For drug-related lactic acidosis, it is often necessary to discontinue the suspected drug [9]. The use of sodium bicarbonate remains inconclusive, and individualized treatment is recommended.

## Case presentation

A 13-day-old male infant, presented with jaundice for 12 days, which gradually increased with an average transcutaneous bilirubin of 15 mg/dL. In the past two days, the infant had a slight bleeding from the navel. The umbilical discharge culture identified methicillin-resistant* Staphylococcus aureus *(MRSA). The infant was admitted with a preliminary diagnosis of neonatal omphalitis and neonatal hyperbilirubinemia. The infant was born at 39 weeks, with an Apgar score of 10 at one minute and a birth weight of 3.02 kg. The infant regularly took vitamin AD 1500 IU:500 IU once daily, with normal breastfeeding and bowel movements.

The physical examination revealed a temperature of 37.1°C, pulse of 142 beats per minute, respiration of 47 breaths per minute, and weight of 3.56 kg. The total bilirubin was 264.0 μmol/L (Table 1). Venous blood gas analysis showed a pH of 7.30 and lactic acid of 5.3 mmol/L (Table 2). The white blood cell count was 7.48×10^9^/L, C-reactive protein (CRP) was less than 0.5 mg/L, and procalcitonin (PCT) was 0.16 ng/mL (Tables 3-4).

**Table 1:** Blood glucose and bilirubin

Item	Day 1	Day 5	Day 6	Reference range	Unit
Glucose	4.40	/	5.30	3.89-6.11	mmol/L
Total bilirubin	264.0	/	/	0.0-23.0	μmol/L
Conjugated bilirubin	10.0	/	/	0.0-4.0	μmol/L
Transcutaneous bilirubin	17.2	7.6	6.6	<12.9	mg/dl

**Table 2:** Blood gas analysis Na^+^: Sodium; Ca^+^: Calcium: K^+^: Potassium; Cl^-^: Chloride; HCO_3_^-^: Bicarbonate

Item	Day 1	Day 6	Day 6 ( FiO_2_=100%)	Day 7	Reference range	Unit
pH	7.30	6.58	7.01	7.22	7.35-7.45	
Lactic acid	5.3	17	19	9.9	0.5-1.6	mmol/L
Venous oxygen saturation	65	/	/	/	60-80	%
Hemoglobin	154	85	98	133	120-175	g/L
Hematocrit	44	26	30	38	42-49	%
Venous oxygen partial pressure	49	/	/	/	37-43	mmHg
Arterial oxygen partial pressure	/	26	84	65	83-108	mmHg
Venous carbon dioxide partial pressure	45	/	/	/	40-50	mmHg
Arterial carbon dioxide partial pressure	/	123	17	43	35-45	mmHg
Na^+^ concentration	134	133	126	131	136-146	mmol/L
K^+^ concentration	4.6	5.9	5.6	5.4	3.5-5.0	mmol/L
Ca^2+^ concentration	1.36	0.89	1.01	1.37	1.15-1.29	mmol/L
Cl^-^ concentration	/	/	99	/	98-106	mmol/L
Base excess	-2.1	-26.2	-25	-9.8	-3-3	mmol/L
Standard base excess	-1.3	-26.8	-26.8	-10.1	-3-3	mmol/L
HCO_3_^-^ concentration	25.1	11.4	4.3	17.6	22-27	mmol/L
Standard HCO_3_^-^ concentration	20.9	5.3	6.9	17	22-27	mmol/L
Anion gap（K^+^）	/	27	29	/	12-16	mmol/L

**Table 3:** Complete blood count and CRP CRP: C-reactive protein

Item	Day 1	Day 4	Reference range	Unit
White blood cell count	7.48	6.42	3.50-9.50	10^9^/L
Neutrophil count	3.19	1.69	1.80-6.30	10^9^/L
Neutrophil percentage	42.7	26.4	40.0-75.0	%
Lymphocyte count	3.58	3.99	1.10-3.20	10^9^/L
Lymphocyte percentage	47.8	62.1	20.0-50.0	%
Monocyte count	0.55	0.49	0.10-0.60	10^9^/L
Monocyte percentage	7.4	7.7	3.0-10.0	%
Eosinophil count	0.14	0.22	0.02-0.52	10^9^/L
Eosinophil percentage	1.8	3.4	0.4-8.0	%
Basophil count	0.02	0.03	0.00-0.06	10^9^/L
Basophil percentage	0.3	0.4	0.0-1.0	%
Hemoglobin	122	105	130.0-175.0	g/L
Red blood cell count	3.62	3.20	4.30-5.80	10^12^/L
Hematocrit	33.7	30.1	40.0-50.0	%
Mean corpuscular volume	93.2	94.2	82.0-100.0	fL
Mean corpuscular hemoglobin	33.8	32.9	27.0-34.0	pg
Mean corpuscular hemoglobin concentration	363	349	316-354	g/L
Red cell distribution width standard deviation	50.8	47.6	35.0-56.0	%
Red cell distribution width coefficient of variation	12.8	13.5	11.5-14.5	%
Platelet count	460	408	125-350	10^9^/L
Mean platelet volume	9.5	11.0	6.0-11.5	fL
Platelet distribution width	16.5	16.1	9.0-20.0	%
Plateletcrit	0.44	0.45	0.10-0.28	%
Reticulocyte	0.008	/	0.005-0.015	
CRP	<0.50	0.41	0-5	mg/L

**Table 4:** Procalcitonin

Item	Day 1	Day 5	Reference range	Unit
Procalcitonin	0.16	0.12	0.00-0.05	ng/mL

The umbilical discharge culture showed growth of MRSA, which was sensitive to vancomycin, linezolid, clarithromycin, gentamicin, tigecycline, and trimethoprim-sulfamethoxazole (TMP-SMZ). Linezolid was prescribed for this child due to concerns about ototoxicity and nephrotoxicity of vancomycin and gentamicin. Tigecycline is not recommended for children under eight years of age. Clarithromycin and TMP-SMZ were not selected because no intravenous formulations were available for neonates. The infant was treated with linezolid 0.035 g q8h, intravenous infusion rate 15 mL/h, and blue light phototherapy. During the treatment, the jaundice and infection improved, with no other discomfort.

**Figure 1:**
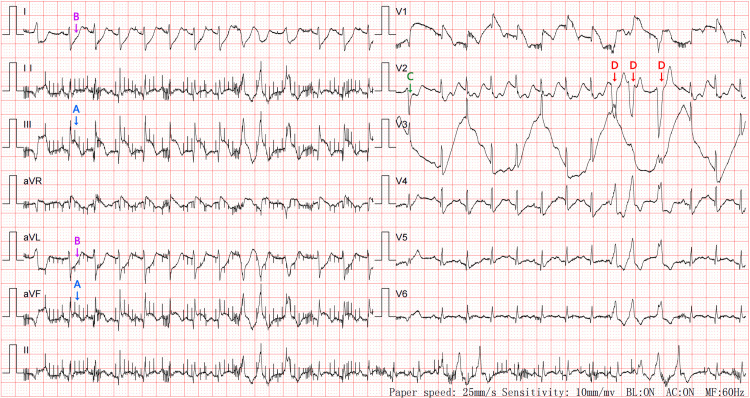
Electrocardiogram after cardiopulmonary resuscitation After successful cardiopulmonary resuscitation, the electrocardiogram showed sinus rhythm, frequent premature ventricular contractions (C), and short bursts of ventricular tachycardia (D). The ST segment was elevated by 0.20-0.35 mV in lead III and lead aVF (A), and depressed by 0.35 mV in lead I and lead aVL (B).

On the morning of the sixth day of hospitalization, the infant suddenly developed groaning and cyanosis, without nausea, vomiting, or diarrhea. Oxygen saturation rapidly dropped to 60% and the heart rate was 50-60 beats per minute. The infant was treated with intravenous epinephrine, external cardiac compression, and endotracheal intubation connected to a ventilator for assisted ventilation. After resuscitation, the infant regained spontaneous breathing, with a frequency of 40-50 breaths per minute, a heart rate of 120-160 beats per minute, and transcutaneous oxygen saturation of 95%-100%. Arterial blood gas (ABG) analysis during this period showed a pH of 6.58, arterial oxygen partial pressure (PaO_2_) of 26 mmHg, arterial carbon dioxide partial pressure (PaCO_2_) of 123 mmHg, lactic acid of 17.0 mmol/L (Table 2), blood glucose of 5.30 mmol/L (Table 1). Severe acidosis was indicated, and the infant was treated with intravenous sodium bicarbonate injection. The electrocardiogram showed frequent ventricular premature beats, short runs of ventricular tachycardia, and ST-T changes (Figure 1). Chest X-ray showed no significant abnormalities, while brain ultrasound indicated cerebral edema. (The examinations were performed at an external medical facility; however, due to unforeseen circumstances, we have been unable to secure the necessary authorization to present the images).

Linezolid was discontinued, and lidocaine and mannitol injection was used. On the seventh day of hospitalization, with ventilator-assisted ventilation, ABG showed a pH of 7.22, PaO_2_ of 65 mmHg, PaCO_2_ of 43 mmHg, and lactic acid of 9.9 mmol/L (Table 2). The family refused any medical intervention, and the infant died after the cessation of ventilator-assisted ventilation.

The autopsy showed findings consistent with cardiac and respiratory arrest, myocardial injury after cardiopulmonary resuscitation, acute necrotizing enterocolitis, and ischemic encephalopathy, leading to multiple organ failure. Genetic testing indicated no inherited metabolic diseases, including mitochondrial diseases.

## Discussion

The etiology of metabolic acidosis in children involves the accumulation of hydrogen (H^+^) ions, such as excessive endogenous acid production, excessive exogenous acid intake, insufficient renal excretion, loss of bicarbonate (HCO_3_^-^) due to diarrhea, loss of intestinal or pancreatic fluids, or dilution by fluids. The infant in this case had normal milk intake and normal bowel movements, without significant fluid replacement, leading to the consideration of excessive endogenous lactate production. Through Naranjo's adverse drug reaction probability scale, it is possible that linezolid was the cause. The mechanism by which linezolid induces lactic acidosis may be related to mitochondrial toxicity. Linezolid acts on bacterial 23S rRNA, which is structurally similar to human mitochondrial 16S RNA, thereby disrupting mitochondrial protein synthesis [5]. Reduced levels of mitochondrial DNA and impaired oxidative phosphorylation lead to a shift from aerobic to anaerobic metabolism, resulting in the production of large amounts of lactate and ultimately causing lactic acidosis [6]. Mitochondrial DNA A2706G polymorphism may be associated with linezolid-induced lactic acidosis [10,11]. During the use of linezolid, there were no adverse symptoms and genetic testing results were normal, while sudden cardiac and respiratory arrest appeared with lactate levels of 17 mmol/L. This suggests that during the treatment, it is not sufficient to rely solely on symptoms and genetic conditions for judgment; ABG monitoring is also necessary for some patients.

The infant also had respiratory acidosis and respiratory failure, and it could not be ruled out that neurological dysfunction led to secondary lactic acidosis due to respiratory and cardiac arrest. Sudden death in infants under one year of age, with no identifiable cause after a comprehensive assessment, is known as sudden infant death syndrome (SIDS). Risk factors for SIDS include prone sleep position, intercurrent illness, male gender, and prematurity. The infant in this case was male and had jaundice and infection, which are high-risk factors for SIDS. The key factor in triggering SIDS is brainstem abnormalities related to the neural regulation of cardiopulmonary control or delayed maturation, which is associated with abnormalities in 5-hydroxytryptamine (5-HT) transmission, characterized by reduced binding of 5-HT receptors, especially in male infants, consistent with the predominance of male infants in SIDS cases [12-14]. Linezolid, as a monoamine oxidase inhibitor, can inhibit the metabolism of monoamines, leading to 5-HT syndrome, characterized by fever, agitation, restlessness, hypertension, vomiting, and diarrhea. However, whether linezolid affects brainstem 5-HT transmission requires further research.

Omphalitis is a polymicrobial infection. The main pathogens include *Staphylococcus aureus*, *Streptococcus pyogenes*, and gram-negative bacteria, including *Escherichia coli*, *Klebsiella pneumoniae*, and *Proteus mirabilis*. Antibiotic treatment targeting both gram-positive and gram-negative bacteria is necessary. For those with obvious pus, umbilical diffusion, and systemic symptoms, vancomycin is the preferred treatment for MRSA infection. Linezolid has serious adverse reactions such as lactic acidosis, 5-HT syndrome, and hematologic toxicity, which should be used with caution. For patients with mild symptoms, some clinicians also take topical treatment such as alcohol and mupirocin, but there is insufficient evidence to support these treatments. In this case, the child had no systemic symptoms such as fever, feeding difficulty, or lethargy, just had a slight bleeding from the navel with an increase of PCT and lactic acid level. Topical treatment or empirical treatment with anti-staphylococcal penicillin could be attempted at the beginning without secretion culture. However, since no other etiologies such as renal excretion disorder, diabetic ketoacidosis, genetic metabolic disease were found, lactic acidosis may be the only manifestation of serious infection and its complications.

## Conclusions

In this case, the infant had no congenital genetic abnormalities. During the use of linezolid, typical symptoms of acidosis did not manifest, yet a sudden cardiac and respiratory arrest occurred. This suggests that during the administration of linezolid, especially in neonates, it is important to monitor blood gas analysis. When treating systemic infections such as MRSA, vancomycin is generally recommended as the first-line treatment. Particular caution should be exercised when selecting linezolid for infants and young children to avoid prolonged and high-dose use. In the event of adverse reactions, the medication should be discontinued immediately, and symptomatic treatment measures should be taken.

## References

[1] Santini A, Ronchi D, Garbellini M, Piga D, Protti A (2017). Linezolid-induced lactic acidosis: the thin line between bacterial and mitochondrial ribosomes. Expert Opin Drug Saf.

[2] Gatti M, Fusaroli M, Raschi E, Moretti U, Poluzzi E, De Ponti F (2021). Serious adverse events with tedizolid and linezolid: pharmacovigilance insights through the FDA adverse event reporting system. Expert Opin Drug Saf.

[3] Im JH, Baek JH, Kwon HY, Lee JS (2015). Incidence and risk factors of linezolid-induced lactic acidosis. Int J Infect Dis.

[4] Dai Y, Wang Y, Zeng Y, Zhang C, Zhou Z, Shi D (2020). Linezolid and the risk of lactic acidosis: data mining and analysis of the FDA adverse event reporting system. J Clin Pharm Ther.

[5] Narita M, Tsuji BT, Yu VL (2007). Linezolid-associated peripheral and optic neuropathy, lactic acidosis, and serotonin syndrome. Pharmacotherapy.

[6] Wiener M, Guo Y, Patel G, Fries BC (2007). Lactic acidosis after treatment with linezolid. Infection.

[7] Mao Y, Dai D, Jin H, Wang Y (2018). The risk factors of linezolid-induced lactic acidosis: a case report and review. Medicine (Baltimore).

[8] Filho RR, Rocha LL, Corrêa TD, Pessoa CM, Colombo G, Assuncao MS (2016). Blood lactate levels cutoff and mortality prediction in sepsis-time for a reappraisal? A retrospective cohort study. Shock.

[9] Smith ZR, Horng M, Rech MA (2019). Medication-induced hyperlactatemia and lactic acidosis: a systematic review of the literature. Pharmacotherapy.

[10] Palenzuela L, Hahn NM, Nelson RP Jr (2005). Does linezolid cause lactic acidosis by inhibiting mitochondrial protein synthesis?. Clin Infect Dis.

[11] Velez JC, Janech MG (2010). A case of lactic acidosis induced by linezolid. Nat Rev Nephrol.

[12] Duncan JR, Paterson DS, Hoffman JM (2010). Brainstem serotonergic deficiency in sudden infant death syndrome. JAMA.

[13] Machaalani R, Say M, Waters KA (2009). Serotoninergic receptor 1A in the sudden infant death syndrome brainstem medulla and associations with clinical risk factors. Acta Neuropathol.

[14] Paterson DS, Trachtenberg FL, Thompson EG (2006). Multiple serotonergic brainstem abnormalities in sudden infant death syndrome. JAMA.

